# 4-(All­yloxy)benzohydrazide

**DOI:** 10.1107/S2414314622011956

**Published:** 2023-01-06

**Authors:** Sultana Shakila Khan, Md. Belayet Hossain Howlader, Ryuta Miyatake, Md. Chanmiya Sheikh, Ennio Zangrando

**Affiliations:** a Department of Chemistry, Rajshahi University, Rajshahi-6205, Bangladesh; b Center for Environmental Conservation and Research Safety, University of Toyama, 3190 Gofuku, Toyama, 930-8555, Japan; c Department of Applied Science, Faculty of Science, Okayama University of Science, Japan; d Department of Chemical and Pharmaceutical Science, University of Trieste, Italy; University of Aberdeen, United Kingdom

**Keywords:** crystal structure, all­yl, benzohydrazide

## Abstract

The non-H atoms of the title compound, C_10_H_12_N_2_O_2_, are approximately coplanar with the exception of those at the ends: the terminal allyl carbon atom and terminal hydrazide nitro­gen atom are displaced from the mean plane by 0.67 (2) and 0.20 (2) Å, respectively. In the crystal, the mol­ecules are linked by N—H⋯O and N—H⋯N hydrogen bonds, which give rise a two-dimensional network propagating in the (001) plane.

## Abstract 2

In the crystal of the title hydrazide, the mol­ecules are linked into (001) sheets by N—H⋯N and N—H⋯O hydrogen bonds.

## Graphical abstract

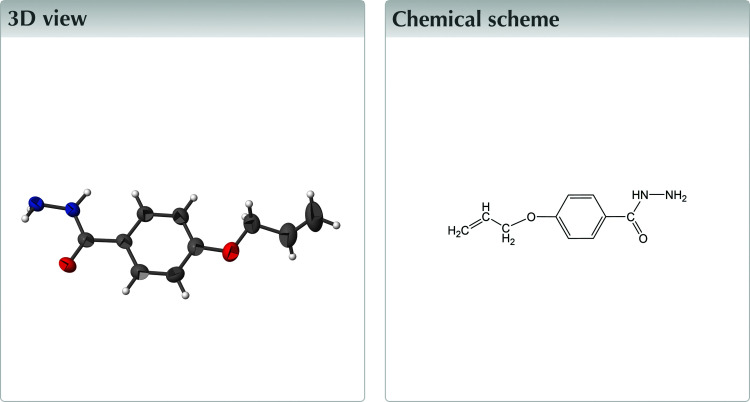

## Structure description

**Figure 1:**
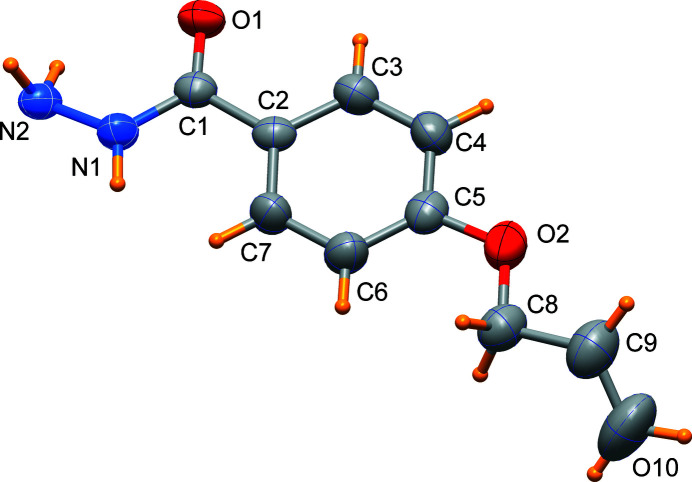
The mol­ecular structure of the title compound showing 50% displacement ellipsoids.

Hydrazides containing an *R*—C(=O)—NH—NH_2_ functional group may act as a pharmacophore and present biological activity (see, for example, Joshi *et al.* 2008 ▸). Hydrazide-containing mol­ecules are effective ligands in coordination chemistry (see, for example, Saygıdeğer Demir *et al.*, 2021 ▸). As part of our studies in this area, we now describe the synthesis and structure of the title compound (Fig. 1 ▸).

**Figure 2:**
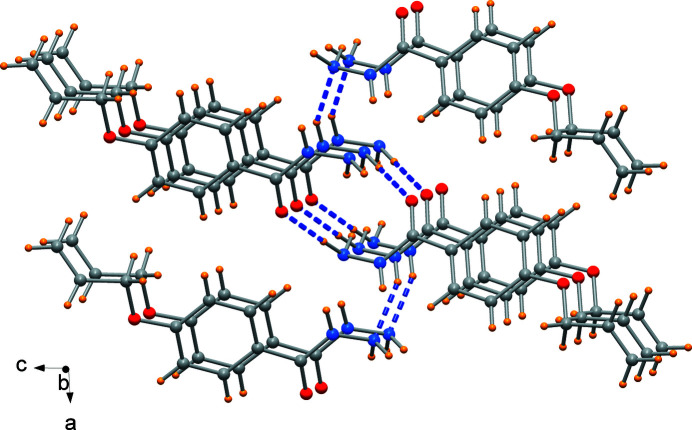
Detail of the crystal packing showing hydrogen-bonding inter­actions as blue dashed lines.

The X-ray diffraction analysis revealed that the non-hydrogen atoms are approximately coplanar with the exception of the terminal atoms, which deviate by 0.67 (2) Å for C10 and 0.20 (2) Å for N2. In the crystal, the mol­ecules are connected by N—H⋯O and N—H⋯N hydrogen bonds involving the carbohydrazide moieties of symmetry-related mol­ecules (Fig. 2 ▸ and Table 1 ▸) that form a two-dimensional network propagating in the *ab* plane. This arrangement favours weak aromatic π–stacking inter­actions of the phenyl rings [centroid-to-centroid distance of 4.092 (3) Å, see Fig. 2 ▸].

## Synthesis and crystallization

A mixture of ethyl-4-hy­droxy­benzoate (8.3 g, 50 mmol) and allyl bromide (6.0 g, 50 mmol) in acetone (100 ml) was refluxed for 20 h over anhydrous potassium carbonate (13.8 g, 100 mmol). The filtrate was collected and the solvent removed *in vacuo*. The resulting colourless oily mass was treated with hydrazine hydrate (5.0 g, 100 mmol) and refluxed for 10 h in ethanol (40 ml). The reaction mixture was left overnight and colourless crystals suitable for X-ray characterization were obtained, filtered off and washed with ethanol. Yield: 7.0 g, (73%), melting point: 355–356 K.

FT–IR (KBr), (cm^−1^): 1650 ν (C=O_ester_), 1621, 1575 ν (C=C), 3328, 3280, 3183 ν (NH—NH_2_).

^1^H NMR(CDCl_3_, 400 MHz), δ: 7.72 (*d*, 2H, C-2,6, *J* = 8.8 Hz), 6.94 (*d*, 2H, C-3,5, *J* = 8.8 Hz), 7.65 (*s*, NH), 4.13 (*s*, 2H, NH_2_), 5.42 (*dq*, H_a_, *J* = 16 Hz, 1.6 Hz), 5.32 (*dq*, H_b_, *J* = 10.4 Hz, 1.2 Hz), 6.05 (*m*, Hc), 4.58 (*dt*, 2H, CH_2_O, *J* = 5.2 Hz, 1.2 Hz).

## Refinement

Crystal data, data collection and structure refinement details are summarized in Table 2 ▸. The absolute structure was indeterminate in the present refinement and the structure was refined as an inversion twin.

## Supplementary Material

Crystal structure: contains datablock(s) I, General. DOI: 10.1107/S2414314622011956/hb4420sup1.cif

Structure factors: contains datablock(s) I. DOI: 10.1107/S2414314622011956/hb4420Isup2.hkl

Supporting information file. DOI: 10.1107/S2414314622011956/hb4420Isup3.cml

CCDC reference: 2210836

Additional supporting information:  crystallographic information; 3D view; checkCIF report

## Figures and Tables

**Table 1:** Hydrogen-bond geometry (Å, °)

*D*—H⋯*A*	*D*—H	H⋯*A*	*D*⋯*A*	*D*—H⋯*A*
N1—H1*A*⋯O1^i^	1.02 (7)	2.00 (7)	3.018 (6)	174 (6)
N1—H1*B*⋯O1^ii^	0.96 (7)	2.50 (6)	3.095 (6)	120 (4)
N2—H2⋯N1^iii^	0.92 (6)	2.11 (6)	2.964 (6)	153 (4)

Symmetry codes: (i) 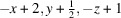 ; (ii) 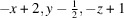 ; (iii) 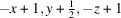 .

**Table 2:** Experimental details

Crystal data	
Chemical formula	C_10_H_12_N_2_O_2_
*M*_r_	192.22
Crystal system, space group	Monoclinic, *P*2_1_
Temperature (K)	263
*a*, *b*, *c* (Å)	5.967 (4), 4.092 (3), 20.358 (14)
β (°)	93.080 (18)
*V* (Å^3^)	496.3 (6)
*Z*	2
Radiation type	Mo *K*α
μ (mm^−1^)	0.09
Crystal size (mm)	0.47 × 0.21 × 0.06

Data collection	
Diffractometer	Rigaku R-AXIS RAPID CCD
Absorption correction	Multi-scan (*ABSCOR*; Rigaku, 1995 ▸)
*T*_min_, *T*_max_	0.299, 0.995
No. of measured, independent and observed [*I* > 2σ(*I*)] reflections	4612, 2075, 1596
*R*_int_	0.073
(sin θ/λ)_max_ (Å^−1^)	0.649

Refinement	
*R*[*F* ^2^ > 2σ(*F* ^2^)], *wR*(*F* ^2^), *S*	0.081, 0.228, 1.05
No. of reflections	2075
No. of parameters	139
No. of restraints	1
H-atom treatment	H atoms treated by a mixture of independent and constrained refinement
Δρ_max_, Δρ_min_ (e Å^−3^)	0.30, −0.30
Absolute structure	Refined as an inversion twin
Absolute structure parameter	0.5

Computer programs: *RAPID-AUTO* (Rigaku, 2010 ▸), *SHELXT2014/5* (Sheldrick, 2015*a* ▸) and *SHELXL2019/2* (Sheldrick, 2015*b* ▸) and *DIAMOND* (Brandenburg & Putz, 1999 ▸).

## full crystallographic data

### Crystal data

**Table:**

C_10_H_12_N_2_O_2_	*F*(000) = 204
*M**_r_* = 192.22	*D*_x_ = 1.286 Mg m^−^^3^
Monoclinic, *P*2_1_	Mo *K*α radiation, λ = 0.71075 Å
*a* = 5.967 (4) Å	Cell parameters from 702 reflections
*b* = 4.092 (3) Å	θ = 3.9–27.4°
*c* = 20.358 (14) Å	µ = 0.09 mm^−^^1^
β = 93.080 (18)°	*T* = 263 K
*V* = 496.3 (6) Å^3^	Plate, colorless
*Z* = 2	0.47 × 0.21 × 0.06 mm

### Data collection

**Table:**

Rigaku R-AXIS RAPID CCD diffractometer	1596 reflections with *I* > 2σ(*I*)
Detector resolution: 10.000 pixels mm^-1^	*R*_int_ = 0.073
ω scans	θ_max_ = 27.5°, θ_min_ = 3.0°
Absorption correction: multi-scan (ABSCOR; Rigaku, 1995)	*h* = −7→7
*T*_min_ = 0.299, *T*_max_ = 0.995	*k* = −5→4
4612 measured reflections	*l* = −26→26
2075 independent reflections	

### Refinement

**Table:**

Refinement on *F*^2^	Hydrogen site location: mixed
Least-squares matrix: full	H atoms treated by a mixture of independent and constrained refinement
*R*[*F*^2^ > 2σ(*F*^2^)] = 0.081	*w* = 1/[σ^2^(*F*_o_^2^) + (0.1107*P*)^2^ + 0.1955*P*] where *P* = (*F*_o_^2^ + 2*F*_c_^2^)/3
*wR*(*F*^2^) = 0.228	(Δ/σ)_max_ = 0.043
*S* = 1.05	Δρ_max_ = 0.30 e Å^−^^3^
2075 reflections	Δρ_min_ = −0.30 e Å^−^^3^
139 parameters	Absolute structure: Refined as an inversion twin
1 restraint	Absolute structure parameter: 0.5
Primary atom site location: dual	

### Special details

**Table:**

Geometry. All esds (except the esd in the dihedral angle between two l.s. planes) are estimated using the full covariance matrix. The cell esds are taken into account individually in the estimation of esds in distances, angles and torsion angles; correlations between esds in cell parameters are only used when they are defined by crystal symmetry. An approximate (isotropic) treatment of cell esds is used for estimating esds involving l.s. planes.
Refinement. Refined as a 2-component perfect inversion twin

### Fractional atomic coordinates and isotropic or equivalent isotropic displacement parameters (Å^2^)

**Table:**

	*x*	*y*	*z*	*U*_iso_*/*U*_eq_	
O1	1.0223 (5)	0.0117 (11)	0.59637 (15)	0.0501 (10)	
O2	0.6099 (6)	0.6905 (12)	0.84332 (16)	0.0576 (11)	
N1	0.7352 (6)	0.0258 (13)	0.48583 (18)	0.0420 (10)	
H1A	0.810 (10)	0.20 (2)	0.459 (3)	0.070 (18)*	
H1B	0.828 (9)	−0.162 (18)	0.495 (3)	0.053 (15)*	
N2	0.6987 (6)	0.1941 (12)	0.54599 (17)	0.0422 (10)	
H2	0.568 (9)	0.310 (17)	0.551 (2)	0.053 (15)*	
C1	0.8432 (7)	0.1605 (13)	0.5991 (2)	0.0381 (10)	
C2	0.7721 (7)	0.3101 (12)	0.6615 (2)	0.0368 (10)	
C3	0.9130 (7)	0.2748 (14)	0.7179 (2)	0.0466 (13)	
H3	1.048347	0.163742	0.715485	0.056*	
C4	0.8538 (8)	0.4031 (15)	0.7774 (2)	0.0512 (14)	
H4	0.948590	0.375286	0.814732	0.061*	
C5	0.6535 (8)	0.5734 (13)	0.7817 (2)	0.0450 (12)	
C6	0.5104 (9)	0.6088 (14)	0.7266 (2)	0.0499 (14)	
H6	0.374650	0.718525	0.729247	0.060*	
C7	0.5717 (8)	0.4782 (15)	0.6669 (2)	0.0476 (13)	
H7	0.475950	0.504343	0.629677	0.057*	
C8	0.4030 (9)	0.8680 (17)	0.8490 (2)	0.0558 (14)	
H8A	0.275903	0.726145	0.838444	0.067*	
H8B	0.396374	1.050785	0.818586	0.067*	
C9	0.3954 (12)	0.9884 (19)	0.9178 (3)	0.0743 (19)	
H9	0.525899	1.076453	0.937629	0.089*	
C10	0.2180 (15)	0.978 (3)	0.9519 (3)	0.105 (3)	
H10A	0.084985	0.891368	0.933402	0.126*	
H10B	0.223805	1.057220	0.994739	0.126*	

### Atomic displacement parameters (Å^2^)

**Table:**

	*U*^11^	*U*^22^	*U*^33^	*U*^12^	*U*^13^	*U*^23^
O1	0.0359 (16)	0.058 (2)	0.0565 (18)	0.0103 (18)	0.0023 (12)	−0.0034 (17)
O2	0.064 (2)	0.064 (3)	0.0447 (18)	−0.003 (2)	0.0052 (14)	−0.0052 (17)
N1	0.033 (2)	0.045 (3)	0.048 (2)	0.0006 (19)	0.0049 (14)	−0.0047 (19)
N2	0.0352 (19)	0.048 (3)	0.0436 (19)	0.007 (2)	0.0032 (14)	−0.0050 (17)
C1	0.034 (2)	0.035 (2)	0.046 (2)	−0.004 (2)	0.0044 (16)	0.0039 (19)
C2	0.032 (2)	0.031 (3)	0.048 (2)	−0.0047 (19)	0.0051 (15)	0.0019 (18)
C3	0.041 (2)	0.047 (3)	0.052 (3)	0.001 (2)	−0.0016 (18)	0.002 (2)
C4	0.046 (3)	0.061 (4)	0.046 (2)	−0.005 (3)	−0.0056 (19)	0.000 (2)
C5	0.050 (3)	0.039 (3)	0.046 (2)	−0.010 (2)	0.0081 (18)	0.000 (2)
C6	0.047 (3)	0.055 (4)	0.048 (2)	0.008 (3)	0.006 (2)	0.001 (2)
C7	0.044 (3)	0.055 (4)	0.044 (2)	0.009 (3)	−0.0007 (17)	0.003 (2)
C8	0.061 (3)	0.050 (3)	0.058 (3)	−0.004 (3)	0.014 (2)	−0.007 (2)
C9	0.094 (4)	0.066 (5)	0.065 (3)	−0.006 (4)	0.020 (3)	−0.009 (3)
C10	0.131 (6)	0.117 (8)	0.072 (4)	0.017 (7)	0.046 (4)	−0.006 (5)

### Geometric parameters (Å, º)

**Table:**

O1—C1	1.234 (6)	C4—C5	1.390 (7)
O2—C5	1.380 (6)	C4—H4	0.9300
O2—C8	1.442 (7)	C5—C6	1.381 (7)
N1—N2	1.432 (5)	C6—C7	1.395 (7)
N1—H1A	1.02 (7)	C6—H6	0.9300
N1—H1B	0.96 (7)	C7—H7	0.9300
N2—C1	1.352 (6)	C8—C9	1.488 (8)
N2—H2	0.92 (6)	C8—H8A	0.9700
C1—C2	1.493 (6)	C8—H8B	0.9700
C2—C3	1.393 (6)	C9—C10	1.297 (9)
C2—C7	1.389 (6)	C9—H9	0.9300
C3—C4	1.383 (7)	C10—H10A	0.9300
C3—H3	0.9300	C10—H10B	0.9300
			
C5—O2—C8	116.8 (4)	C6—C5—C4	119.8 (4)
N2—N1—H1A	102 (4)	O2—C5—C4	115.8 (4)
N2—N1—H1B	109 (3)	C5—C6—C7	119.2 (5)
H1A—N1—H1B	114 (5)	C5—C6—H6	120.4
C1—N2—N1	121.0 (4)	C7—C6—H6	120.4
C1—N2—H2	118 (3)	C2—C7—C6	121.7 (4)
N1—N2—H2	121 (3)	C2—C7—H7	119.1
O1—C1—N2	122.1 (4)	C6—C7—H7	119.1
O1—C1—C2	121.8 (4)	O2—C8—C9	108.1 (5)
N2—C1—C2	116.1 (4)	O2—C8—H8A	110.1
C3—C2—C7	118.1 (4)	C9—C8—H8A	110.1
C3—C2—C1	118.2 (4)	O2—C8—H8B	110.1
C7—C2—C1	123.7 (4)	C9—C8—H8B	110.1
C4—C3—C2	120.7 (5)	H8A—C8—H8B	108.4
C4—C3—H3	119.7	C10—C9—C8	124.0 (8)
C2—C3—H3	119.7	C10—C9—H9	118.0
C3—C4—C5	120.4 (4)	C8—C9—H9	118.0
C3—C4—H4	119.8	C9—C10—H10A	120.0
C5—C4—H4	119.8	C9—C10—H10B	120.0
C6—C5—O2	124.3 (5)	H10A—C10—H10B	120.0
			
N1—N2—C1—O1	7.3 (8)	C8—O2—C5—C4	180.0 (5)
N1—N2—C1—C2	−171.9 (4)	C3—C4—C5—C6	1.4 (8)
O1—C1—C2—C3	−0.8 (7)	C3—C4—C5—O2	179.8 (5)
N2—C1—C2—C3	178.5 (5)	O2—C5—C6—C7	−179.6 (5)
O1—C1—C2—C7	−179.7 (5)	C4—C5—C6—C7	−1.3 (8)
N2—C1—C2—C7	−0.5 (7)	C3—C2—C7—C6	−0.1 (8)
C7—C2—C3—C4	0.2 (7)	C1—C2—C7—C6	178.8 (5)
C1—C2—C3—C4	−178.8 (5)	C5—C6—C7—C2	0.7 (8)
C2—C3—C4—C5	−0.8 (8)	C5—O2—C8—C9	−176.7 (5)
C8—O2—C5—C6	−1.7 (8)	O2—C8—C9—C10	−137.3 (8)

### Hydrogen-bond geometry (Å, º)

**Table:**

*D*—H···*A*	*D*—H	H···*A*	*D*···*A*	*D*—H···*A*
N1—H1*A*···O1^i^	1.02 (7)	2.00 (7)	3.018 (6)	174 (6)
N1—H1*B*···O1^ii^	0.96 (7)	2.50 (6)	3.095 (6)	120 (4)
N2—H2···N1^iii^	0.92 (6)	2.11 (6)	2.964 (6)	153 (4)

Symmetry codes: (i) −*x*+2, *y*+1/2, −*z*+1; (ii) −*x*+2, *y*−1/2, −*z*+1; (iii) −*x*+1, *y*+1/2, −*z*+1.
